# Decomposition of Organic Perovskite Precursors on MoO_3_: Role of Halogen and Surface Defects

**DOI:** 10.1021/acsami.1c20847

**Published:** 2022-02-02

**Authors:** Sofia Apergi, Christine Koch, Geert Brocks, Selina Olthof, Shuxia Tao

**Affiliations:** † Materials Simulation and Modelling, Department of Applied Physics, Eindhoven University of Technology, P.O. Box 513, 5600 MB Eindhoven, The Netherlands; ‡ Center for Computational Energy Research, Department of Applied Physics, Eindhoven University of Technology, P.O. Box 513, 5600 MB Eindhoven, The Netherlands; § Department of Chemistry, University of Cologne, Greinstraße 4-6, 50939 Cologne, Germany; ∥ Computational Materials Science, Faculty of Science and Technology and MESA+, Institute for Nanotechnology, University of Twente, P.O. Box 217, 7500 AE Enschede, The Netherlands

**Keywords:** perovskite, metal oxide, DFT, photoelectron spectroscopy, stability

## Abstract

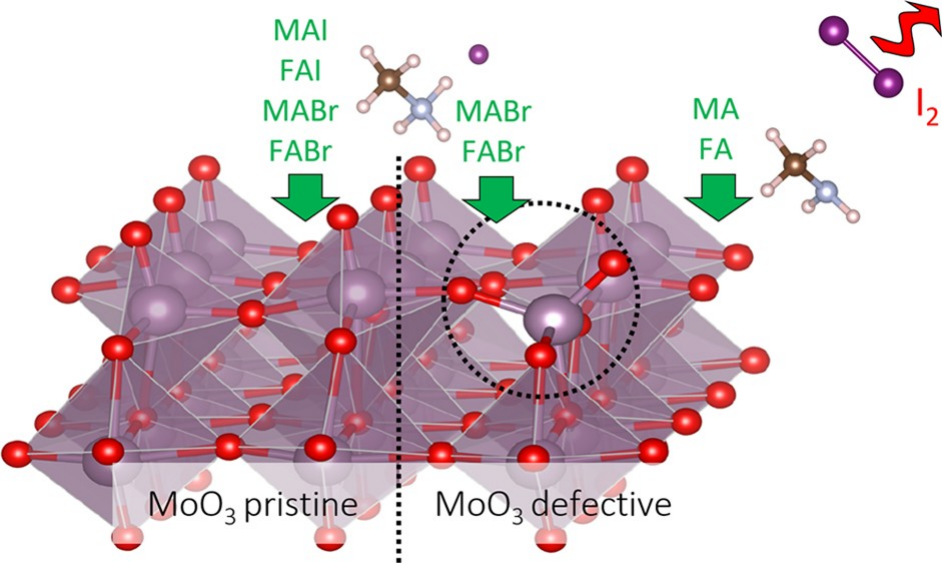

Despite the rapid progress in perovskite solar cells, their commercialization is still hindered by issues regarding long-term stability, which can be strongly affected by metal oxide-based charge extraction layers next to the perovskite material. With MoO_3_ being one of the most successful hole transport layers in organic photovoltaics, the disastrous results of its combination with perovskite films came as a surprise but was soon attributed to severe chemical instability at the MoO_3_/perovskite interface. To discover the atomistic origin of this instability, we combine density functional theory (DFT) calculations and X-ray photoelectron spectroscopy (XPS) measurements to investigate the interaction of MoO_3_ with the perovskite precursors MAI, MABr, FAI, and FABr. From DFT calculations we suggest a scenario that is based upon oxygen vacancies playing a key role in interface degradation reactions. Not only do these vacancies promote decomposition reactions of perovskite precursors, but they also constitute the reaction centers for redox reactions leading to oxidation of the halides and reduction of Mo. Specifically iodides are proposed to be reactive, while bromides do not significantly affect the oxide. XPS measurements reveal a severe reduction of Mo and a loss of the halide species when the oxide is interfaced with I-containing precursors, which is consistent with the proposed scenario. In line with the latter, experimentally observed effects are much less pronounced in case of Br-containing precursors. We further find that the reactivity of the MoO_3_ substrate can be moderated by reducing the number of oxygen vacancies through a UV/ozone treatment, though it cannot be fully eliminated.

## 1 Introduction

Recently, metal halide perovskites have drawn unprecedented attention, due to their highly attractive properties, such as tunable band gaps and high carrier mobility that make them ideal for a range of optoelectronic applications.^1−3^ Within just a few years, perovskite solar cells (PSCs) have achieved efficiencies of >25%, a progress that took decades for other photovoltaic technologies.^4−6^ In early studies, the material of choice was MAPbI_3_,^7,8^ while in the last years, more complex mixed perovskite compositions have been the focus of the research community.^5,9^ Nevertheless, there are issues that remain to be addressed for the commercialization of PSCs, most notably the long-term stability, especially when devices are exposed to humidity, light, elevated temperatures, or oxygen.^10−16^

In addition to the active perovskite material, PSCs typically contain charge extraction layers, that selectively transport charges to the contacts.^17^ At such an interface, it is obviously important to ensure an appropriate alignment of energy levels to minimize losses in device performance.^18^ Good charge extraction can be achieved by a variety of organic molecules and polymers,^19−21^ metal oxides,^22−24^ or 2D materials.^25^

Over the years, it has become clear that not only the energetic alignment plays a crucial role, but the chemical compatibility also has to be considered. While the contact between perovskites and organic charge extraction layers seems to be benign,^26,27^ strong redox or Lewis acid base reactions with metal oxides have been reported.^27−33^ Since metal oxides are essential in the development of low cost and large area devices, it is of paramount importance to understand the origin of these interface reactions between metal oxides and perovskites.

One notable example of detrimental interface decomposition concerns the material MoO_3_. While in the field of organic photovoltaics MoO_3_ is one of the most successful hole extraction layers,^34−38^ this success could not be replicated in PSCs, as devices with a MAPbI_3_ absorber in contact with MoO_3_ yield very low power conversion efficiencies.^39,40^ While the strong p-doping ability of MoO_3_ is advantageous to facilitate hole extraction from an organic semiconductor layer, this capability to oxidize seems to be detrimental for perovskites. Several X-ray photoelectron spectroscopy (XPS) studies have attempted to elucidate the interaction between MAPbI_3_ and this metal oxide by probing the elemental composition at the interface and found a decomposition of MA or FA molecules, a loss of iodide, and even a loss of lead.^27,41−44^ This is accompanied by changes in the element oxidation states, most prominently the severe reduction of Mo.^27,40−45^ While the previous studies all agree on the deterioration of, both, the MoO_3_ and the perovskite, the underlying reaction mechanisms and reaction routes remain unclear.

Understanding the atomistic origin of the chemical instability could provide valuable insights regarding strategies to prevent or mitigate such issues at the perovskite–metal oxide interface. MoO_3_ is of particular interest to study this interaction as it is known to be an extreme case, so interactions can be studied in detail. Here, to understand the mechanisms for degradation, it is important to analyze which component of the perovskite and which property of the metal oxide surface triggers the reactions. So far, XPS or Raman studies regarding perovskite degradation in contact to MoO_3_ were mostly done using MAPbI_3_.^27,40−43,45^ Therefore, it is not clear whether the instability is inherent to MoO_3_, or if possibly the organic cation or the halide species are responsible. Such investigations can best be done using only the organic cation precursors, as for example shown in studies on other metal oxides such as NiO and TiO_2_.^46,47^ These publications showed that precursor materials undergo similar decomposition reactions compared to the perovskite material.

In this work, we combine density functional theory (DFT) calculations with experimental XPS investigations to shed light on the interaction between MoO_3_ and various organic perovskite precursors AX (A = MA, FA; X = I, Br). From DFT calculations on model MoO_3_ surfaces and precursor molecules we try to identify probable reaction pathways that can degrade the AX precursors and reduce the metal oxide. This allows us to disentangle the influences of organic cations from those of the halide species on this surface. These calculations point toward the high reactivity of iodides as the main cause for interface instability, as they are oxidized by the MoO_3_ substrate while bromides do not significantly react with this metal oxide. The effect of changing the cation is more subtle, where MAI is expected to degrade faster than FAI. The presence of surface oxygen vacancies is, however, vital for these reactions to occur. These mechanisms, suggested by the DFT calculations, are in excellent agreement with experimental observations made by XPS where we monitor changes in oxidation states and surface composition. Importantly, by reducing the number of oxygen vacancies on the MoO_3_ surface via ozone treatment, we manage to reduce the reactivity of MoO_3_ with iodide containing precursors, confirming the key role of oxygen vacancies for the decomposition process.

## 2 Results and Discussion

In the following, we present our findings regarding the interaction between MoO_3_ and the perovskite precursors. In section 2.1, we employ detailed DFT calculations to investigate four possible dissociation and deprotonation reactions of the different precursor molecules in contact to a pristine MoO_3_ surface. Since there is no indication of an increased reactivity at this interface, the calculations are repeated on a surface containing oxygen vacancies, which indeed changes the reaction energies significantly. In addition, two different possible redox reactions are identified, which can lead to a reduction of Mo on the surface. In section 2.2 we compare the reactions suggested by DFT with experiments performed using XPS. Here, the interface composition as well as the oxidation states of the different species can be analyzed and associated with the different reaction pathways.

### 2.1 DFT Calculations

#### Adsorption on Pristine MoO_3_

**Figure 1:**
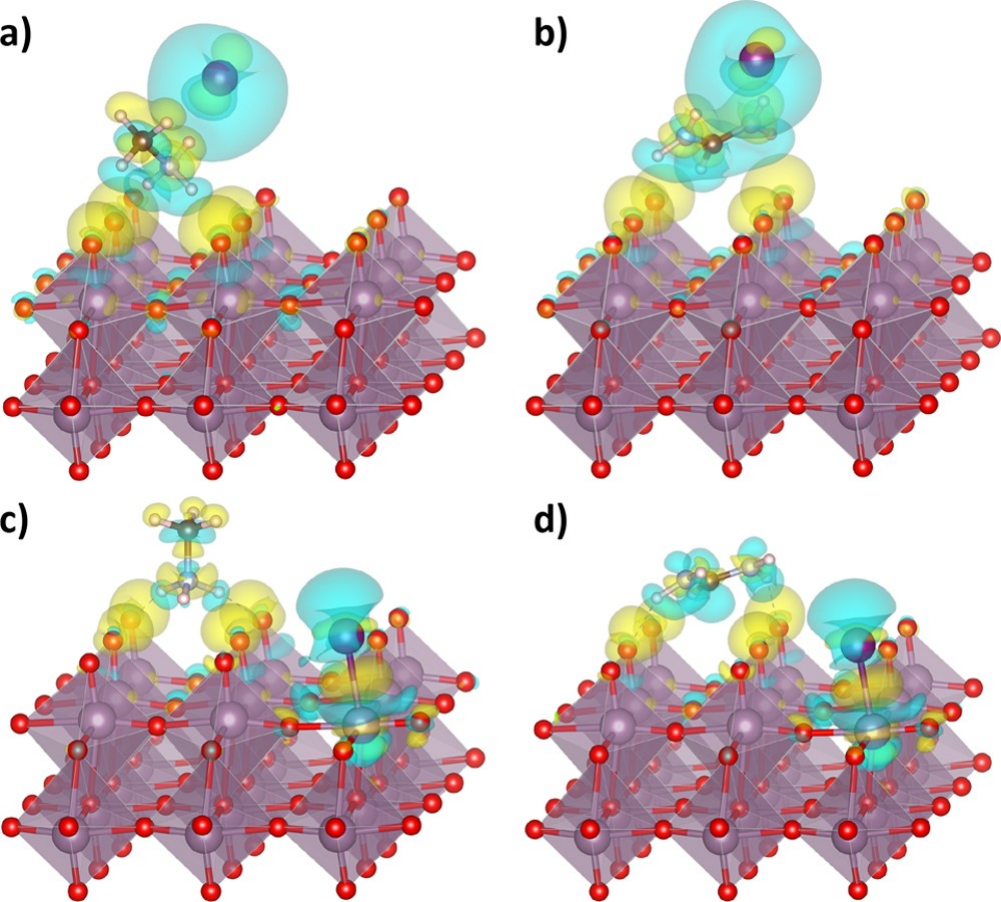
Adsorption geometry of MAI (a) and FAI (b) on the MoO_3_ pristine surface (MABr and FABr adsorb similarly). The yellow and blue colors indicate electron accumulation and depletion regions, respectively, calculated as iso-surfaces of the charge density difference between the adsorbed and the free-standing molecule and surface. (c,d) Adsorption geometries for a defective MoO_3_ surface with one O vacancy. Here, upon adsorption of MAI or FAI, the vacancy is replaced by I.

We investigate the adsorption of AX molecules on the pristine MoO_3_ surface first. As is shown in Figure 1a and b, the halide ions, being negatively charged, are repelled from the negatively charged oxygen atoms that terminate the surface, while the organic cations adsorb on the substrate through hydrogen bonds that are formed between the molecules and the surface oxygen atoms. Adsorption energies can be defined as *E*_ads_ = *E*_MoO3*/*adsorbate_ – *E*_MoO3_ – *E*_adsorbate_, where more negative energies indicate stronger adsorption. As shown in Table 1, calculated adsorption energies range from −0.58 to −0.87 eV, with I-containing molecules exhibiting a noticeably more favorable absorption compared to Br.

**Table 1:** Adsorption Energies and Electron Transfer (Characterized by Net Charge) of AX Precursors to the Pristine and Defective MoO_3_ Surfaces^a^

	pristine MoO_3_				defective MoO_3_			
		net charge (e)				net charge (e)		
adsorbed species	adsorption energy (eV)	A	X	AX	adsorption energy (eV)	A	X	AX
MAI	–0.87	+0.77	–0.32	+0.45	–1.63	+0.82	–0.22	+0.60
FAI	–0.79	+0.79	–0.35	+0.44	–1.30	+0.87	–0.24	+0.63
MABr	–0.62	+0.76	–0.39	+0.37	–1.65	+0.82	–0.29	+0.53
FABr	–0.58	+0.79	–0.42	+0.37	–1.34	+0.87	–0.30	+0.57

a The total net charge of AX in gas phase is 0. The positive net charge of AX adsorbed on oxides indicates electron transfer from AX to the surface.

This trend can be explained by the charges on the molecules, calculated with the DDEC6^48,49^ method, which are also included in Table 1; for a comparison to the gas phase, we refer to Table S1. Here, the electron displacement upon the adsorption of the AX molecules on the oxide surface is an indicator of the electrostatic interaction of the molecules with the oxide. There is a charge displacement from the AX ion pairs to the MoO_3_ substrate, as evidenced by the AX positive net charge in the range of 0.37–0.45e. The larger the charge, the larger the electrostatic interaction between the AX molecule and the substrate and the stronger the adsorption. The slightly more favorable adsorption of the MA containing pairs compared to the FA ones can be explained by the fact that the former molecule has a larger dipole moment than the latter, interacting more strongly with the O-terminated MoO_3_ surface. This effect can also be seen from the slightly shorter and therefore stronger N–H···O hydrogen bonds formed with the MoO_3_ surface, which are listed in Table S2.

The chemical instability at the interface between perovskites and MoO_3_ is often attributed to electron transfer from the perovskites to the oxide and the subsequent reduction of Mo^6+^. However, it is evident from the charge difference plots in Figure 1, that the adsorption of AX on the pristine MoO_3_ surface leaves the oxidation state of the surface Mo atoms unaffected. Instead, the electron displacement from the precursor molecules to the oxide involves the surface O atoms, whose charge becomes more negative (Figure 1a and b).

#### Decomposition on a Pristine MoO_3_

While simple adsorption of the perovskite precursor molecules on the pristine MoO_3_ surface does not have any significant effect on the surface, it might facilitate decomposition of the molecules. To study this, we select two possible reaction paths for MAX and FAX decomposition that have been suggested in the literature,^50,51^ and we examine how adsorption of the molecules and the decomposition products on the surface affects the reaction energies. The reactions considered for MAX are
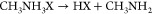
A1and
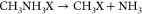
A2For FAX, the reactions are
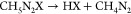
B1and
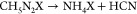
B2A1 and B1 (reaction type 1) are simple proton transfer reactions, corresponding to deprotonation of MA^+^ and FA^+^ ions to form neutral MA and FA molecules, where the proton is used to create a neutral HX molecule. A2 and B2 (reaction type 2) refer to a dissociation of MA or FA that involves breaking a C–N bond.

Possible decomposition reactions of MAI and MABr are reviewed and discussed in great detail in ref (50) where it is argued that the proton transfer reaction (reaction A1) is in competition with C–N bond breakage (reaction A2). In ref (33) it is shown that the presence of a substrate can tip the balance either in favor of the former or of the latter, depending on the substrate. In ref (51), reaction B2 is identified from experiments as a possible decomposition reaction of FAI and FABr.

**Figure 2:**
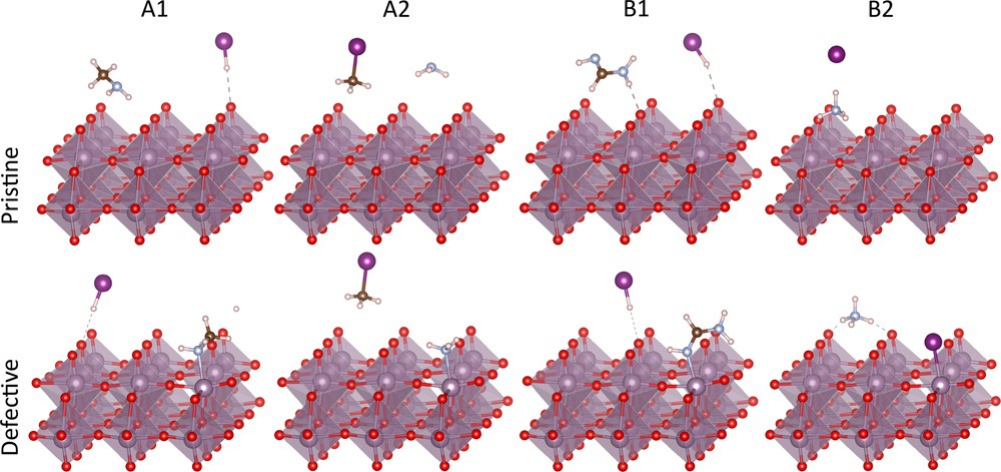
Atomistic representations of the products of reactions A1, A2, B1, and B2 for MAI and FAI adsorbed on a MoO_3_ surface. Top row shoes pristine surface and bottom row adefective surface with one oxygen vacancy (at front right). A similar geometry applies for MABr/FABr. Note that HCN (product of reaction B2) is not presented here as it leaves the surface of MoO_3_. The adsorbed starting reactant molecules are presented in Figure 1.

We define the dissociation energies as *E*_diss_ = *E*_fin_ – *E*_init_, where *E*_fin_ and *E*_init_ are the energies of the reaction product molecules and the starting reactant molecules, respectively, when adsorbed on the surface. The geometries of the reaction products and the dissociation energies are presented in Figure 2 and Table 2, respectively. For comparison, the corresponding dissociation energies for the free-standing molecules are also included in Table 2. Notably, for free-standing molecules all reactions exhibit large positive energies in the range of 0.64 to 1.48 eV, except for the relatively low energies for the dissociation of MAI and MABr (0.24 and 0.34 eV, respectively). This indicates that all molecules are stable, while MA ions are more prone to dissociation than FA ions. This tendency can be explained by the different nature of the C–N bond in MA and FA ions: the former is a single bond (bond order of 1.07) and the latter resembles a delocalized double bond (bond order of 1.55), which is harder to break (Figure S1).

**Table 2:** Reaction Energies of the Deprotonation and Dissociation Reactions of Free-Standing AX Molecules and for Molecules and Reaction Products Adsorbed on Pristine MoO_3_ and a Defective MoO_3_ Surface Containing an Oxygen Vacancy^a^

	free-standing		pristine MoO_3_		defective MoO_3_	
	deprotonation reaction 1	dissociation reaction 2	deprotonation reaction 1	dissociation reaction 2	deprotonation reaction 1	dissociation reaction 2
MAI	0.65	0.24	1.20	0.92	0.43	0.14
FAI	1.08	1.55	1.59	1.37	0.32	0.69
MABr	0.64	0.34	0.92	0.81	0.15	0.01
FABr	1.05	1.48	1.34	1.37	0.05	0.44

a All numbers are in eV.

The deprotonation reaction is also easier for MAX than for FAX molecules, which is consistent with the p*K*_a_ of MA being lower than that of FA (in water 10.66^52^ versus 12.52^53^). It should be also be noted that, as HI is a slightly stronger acid than HBr (p*K*_a_ values in water −10.0 versus −9.0),^54^ one expects the deprotonation reaction for ABr molecules to be slightly easier than for AI molecules. Table 2 shows that this is indeed the case, but the effect for free-standing molecules is very small. The interaction with the substrate plays a larger role, see below.

The positive reaction energies agree with the observed stability of the AX compounds, whose dissociation to gas phase products is entropy-driven, and only occurs at elevated temperatures.^33^ Intriguingly, all reactions seem to be further suppressed on the pristine MoO_3_ surface. This is evidenced by the significantly increased energies for all reactions, now ranging from 0.92 to 1.59 eV. We note that for FAX the dissociation energies are slightly decreased, but they are still much larger than 1 eV, indicating that these reactions are unlikely to occur. The unfavorable decomposition of the AX molecules on the pristine MoO_3_ is related to the small adsorption energies, and thus weaker electrostatic interaction, of the resulting uncharged reaction products (ranging from −0.04 to −0.25 eV) compared to the adsorption of the intact precursors (−0.58 to −0.87 eV), as shown in Tables 1 and S3.

It should be noted that the deprotonation reaction has become somewhat easier for Br containing molecules, as compared to I containing molecules, whereas there is hardly any difference between the two for the free-standing molecules. We believe that this is because the product molecule HBr has a larger dipole moment than HI, which makes its electrostatic interaction with the substrate stronger, and increases its adsorption strength.

For the molecules adsorbed on surfaces it is unlikely that entropy considerations are going to change this picture significantly. At very low concentration of adsorbed molecules, reactions A1–B2 are of course entropy-driven. A simple estimate of the change in mixing entropy per starting molecule for reactions A1–B2 gives Δ*S*_mix_ = −*k*_B_[ln *x* – (2 – 1/*x*)ln(1 – 2*x*) + (1 – 1/*x*)ln(1 – *x*)], with *k*_B_ as the Boltzmann constant and *x* as the fraction of surface sites occupied by adsorbed starting molecules. For instance, at *x* = 1/4 and room temperature, this gives a contribution to the reaction free energy of −*T*_0_Δ*S*_mix_ ≈ −0.05 eV, which is by far insufficient to counteract the large positive dissociation energies.

#### Adsorption and Decomposition on a Defective MoO_3_ Surface

So far, our results indicate that the pristine MoO_3_ surface is not reactive. However, surface defects, such as oxygen vacancies, are expected to be present on any metal oxide at a concentration of a few percent.^55−57^ These vacancies will also be present on MoO_3_, which will be confirmed by XPS measurements shown in section 2.2 below. Accordingly, we continue our DFT study of reactions A1–B2, this time with an O vacancy on the MoO_3_ surface. In this case, the adsorbed AX molecule breaks up into the organic cation A^+^ and the halide anion X^–^ (Figure 1c,d). The organic cation interacts weakly with the surface O atoms, similar as in the case of the pristine surface, but the halide anion takes the place of the O vacancy, where it has a strong interaction with the exposed Mo atom underneath. The result is a notable increase in adsorption energy, which doubles for some of the molecules compared to the pristine surface (Table 1). Differences in adsorption energies come mostly from the choice of organic species. Like we argued for adsorption on the pristine surface, MA has a larger dipole moment and shorter hydrogen bonds to the metal oxide, leading to stronger adsorption compared to FA.

In contrast to the pristine surface, the presence of an O vacancy on the MoO_3_ surface lowers all energies of reactions A1–B2 significantly compared to either the free-standing molecules or the molecules adsorbed on the pristine MoO_3_ surface (Table 2). This happens because all reaction products interact much more strongly with the defective MoO_3_. As shown in Figure 2, the neutral MA, NH_3_, and FA molecules (products of reactions A1, A2, and B1) occupy the position of the O vacancy, acting as Lewis bases with their N atoms forming bonds with the exposed Mo atom underneath. Among the products of reaction B2 is NH_4_X, which splits up into an NH_4_^+^ cation adsorbed on the surface, and an X^–^ anion occupying the position of the O vacancy. As this latter situation is not so different from that of the starting products (Figure 1d), the energy of reaction B2 is not decreased to the same extent as the other reactions.

The adsorption energies of all species involved in reactions A1–B2 are summarized in Table S3. Clearly, in comparison to the pristine MoO_3_ surface, both the deprotonation and the dissociation of AX precursors are promoted in the presence of oxygen vacancies. As discussed above, deprotonation is easier for Br than for I containing species, because the product molecule HBr has a larger dipole moment than HI, making its electrostatic interaction with the substrate stronger, and increasing its adsorption strength. In contrast to the free-standing molecules, deprotonation of FA containing molecules has also become easier than that of MA containing molecules. This reflects the stronger interaction of the neutral (Lewis base) FA molecule with the Mo atom in the substrate that is left exposed by the oxygen vacancy. Dissociation of MA containing species is easier than that of FA containing ones, because of its weaker C–N bond, as discussed above.

Like for the pristine case, we expect entropy effects for these surface reactions to be small. For a fixed O vacancy configuration on the surface, simple mixing entropy for reactions A1, A2, and B1 is absent, as the nitrogen containing product, MA, NH_3_, or FA, always adsorbs at the O vacancy sites. In case of reaction B2 there will be a limited mixing entropy effect as discussed above, which is however insufficient to make this reaction favorable. Nevertheless, reactions with energies ≲0.1 eV, Table 2, may be somewhat stabilized by entropy effects.

In conclusion, adsorption of an AX molecule on a defective MoO_3_ surface results in X^–^ ions occupying O vacancy sites where they bind to Mo atoms. In the case of MAX, there is a possibility that a MA molecule dissociates, leaving NH_3_ to occupy the O vacancy sites. The reaction energy being close to zero suggests that the Lewis base NH_3_ can be easily exchanged with another Lewis base such as a X^–^ ion coming from a second AX molecule. Deprotonation leads to neutral MA or FA molecules occupying O vacancy sites, which, following a similar reasoning, can be exchanged with X^–^.

#### Redox Reaction 1

**Figure 3:**
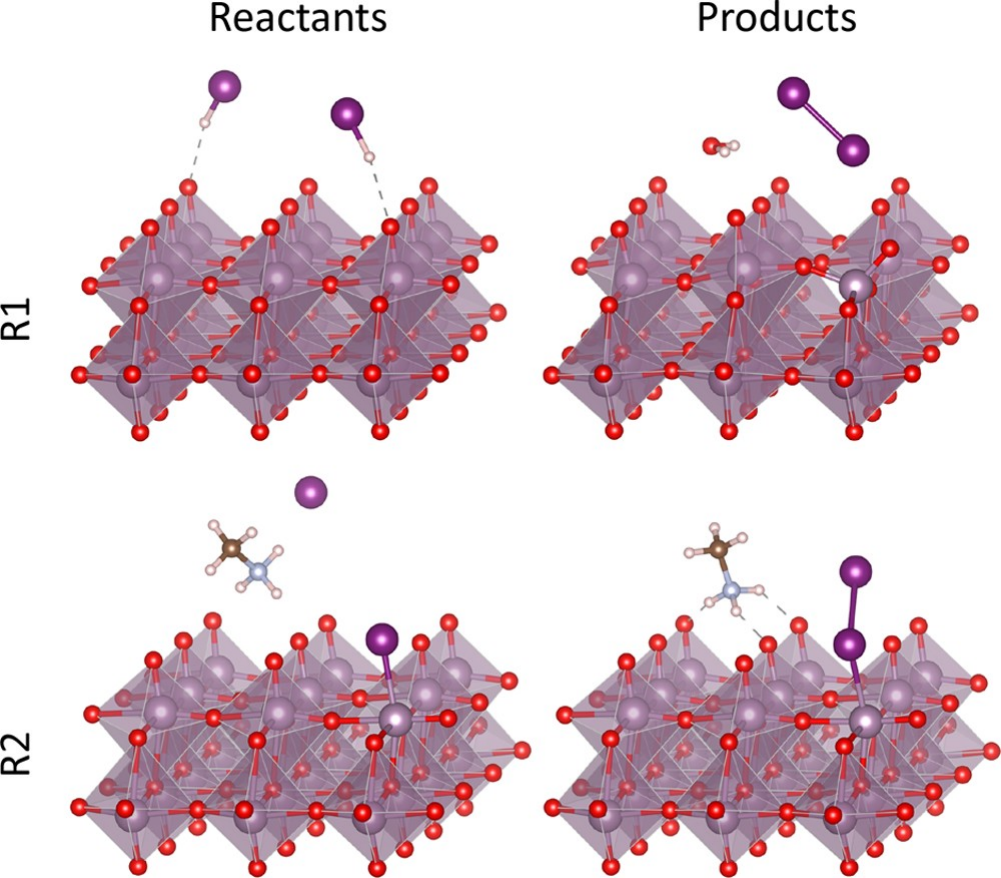
Atomistic representation of redox reaction (R1), oxidizing 2HI to I_2_, creating a H_2_O molecule and a surface oxygen vacancy, and redox reaction (R2), oxidizing one I^–^ adsorbed on an O vacancy position and one I^–^ from an adsorbed MAI molecule, to I_2_.

While the above reactions might lead to precursor decomposition, they do not explain the degradation of MoO_3_ that has been observed experimentally. As MoO_3_ is an oxidizing agent and halide ions can be potentially reduced, this opens possibilities for redox reactions between the metal oxide and the AX adsorption/dissociation products. In Figure 3, we illustrate how HX molecules adsorbed on the surface of MoO_3_ can lead to the formation of X_2_ and the creation of an O vacancy (V_O_), according to the following reaction:
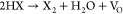
R1As before, we assume that all molecules are adsorbed on the surface; see Figure 3. The calculated reaction energies are −0.11 and +0.80 eV for HI and HBr, respectively. The disparity can be explained from the facts that HI is a somewhat stronger Brønsted acid than HBr (p*K*_a_ HI in water −1.00 versus HBr −9.0),^54^ but, more importantly, reduction of iodides is easier than that of bromides (the standard reduction potential of I_2_ to 2I^–^ is 0.54 V, whereas that for Br_2_ to 2Br^–^ is 1.07 V^52^). Therefore, reaction R1 is likely to happen for HI but unlikely for HBr.

**Figure 4:**
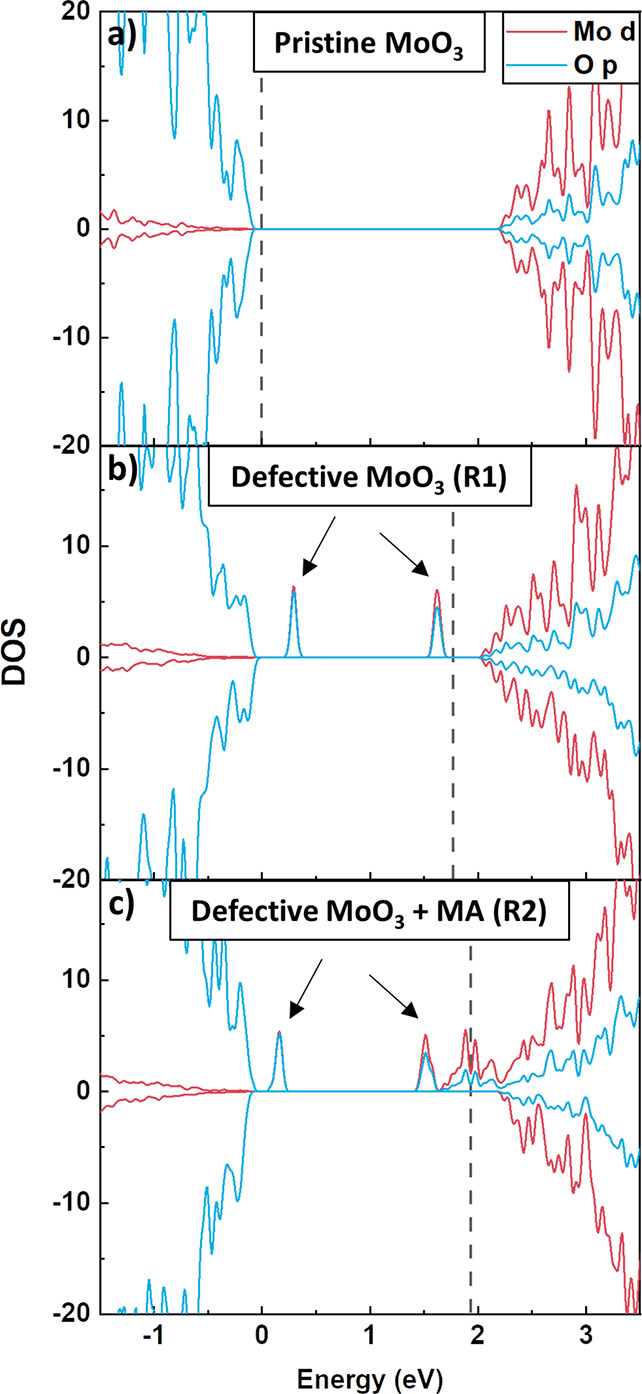
Projected spin polarized electronic density of states (DOS). (a) DOS of a pristine MoO_3_ slab, (b) DOS of defective MoO_3_ with a surface oxygen vacancy created by reaction (R1), and (c) DOS of a defective MoO_3_ after reaction (R2). The zero energy is set at the valence band maximum. The dashed line denotes the highest occupied state.

Notably, reaction R1 can take place without the presence of any oxygen vacancy, instead a vacancy is created in the process. This leads to the reduction of MoO_3_, as can be seen clearly when comparing the calculated density of states (DOS) of a pristine MoO_3_ surface to that of a surface with an O vacancy, as shown in Figure 4. The DOS of a pristine surface (Figure 4a) is typical for a metal oxide semiconductor/insulator, with the top of the valence band being dominated by O p states, and the bottom of the conduction band by Mo d states, respectively, which is consistent with the Mo species being fully oxidized to Mo^6+^. Upon the creation of an oxygen vacancy, we observe the appearance of two peaks within the bandgap, with a mixed Mo–O character (Figure 4b). Judging from the lack of dispersion of these peaks, the corresponding states are localized. It should be noted that, in agreement with ref (58), creation of an O vacancy leads to a sizable local geometry distortion in the lattice. A spatially resolved DOS shows that the two gap states are localized around this geometry distortion (Figure S4), and particularly around two Mo atoms at the center of the distortion. The two gap states are each occupied by a single electron (spin-up, Figure 4b), which in chemical terms corresponds to a reduction of two Mo atoms from Mo^6+^ to Mo^5+^.

This situation described here represents a neutral O vacancy defect, which has two donor levels in the bandgap. Acting as a double donor, the maximum charge of an oxygen vacancy is 2+, which indeed has been found as the most stable charge state in previous work.^58^

#### Redox Reaction 2

As discussed above, the creation of an O vacancy reduces MoO_3_, but there is another process that can even further reduce the defective oxide. The presence of an O vacancy V_O_ on the surface of MoO_3_ leaves a Mo atom exposed and creates an adsorption site for the halogen X of a precursor molecule, X-V_O_, as discussed above (Figure 1c,d). The adsorption of a second AX molecule near this site can trigger a catalytic redox reaction
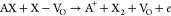
R2as shown in Figure 3. From the reaction energies listed in Table 3, we can see that out of the four precursors, the reaction for MAI is deemed to be most favorable, followed by FAI, whereas the reactions for the Br containing species are less favorable. This finding reflects the facts that iodides are more easily oxidized than bromides, and MA^+^ adsorbs more strongly on the surface than FA^+^ (Table 1). Therefore, we expect the interaction of the defective MoO_3_ surface with MAI to lead to the formation of I_2_. The adsorption energy of I_2_ on the oxide is −0.17 eV (with respect to the surface with a O vacancy and a free molecule). As the condensation energy of a I_2_ molecular solid is ∼0.8 eV/molecule,^59^ it is thus energetically advantageous for the I_2_ molecule to leave the MoO_3_ surface.

**Table 3:** Reaction Energies (eV) of the Two Redox Reactions R1 and R2^a^

R1: 2HX → X_2_ + H_2_O + V_O_		R2: AX + X-V_O_ → A^+^ + X_2_ + V_O_ + *e*	
MAI	–0.11	MAI	–0.11
FAI	–0.11	FAI	+0.10
MABr	+0.80	MABr	+0.21
FABr	+0.80	FABr	+0.39

a X-V_O_ refers to an oxygen vacancy site occupied by a halide.

Redox reaction R2 further reduces the MoO_3_ substrate, which can be seen in the DOSs for MoO_3_ with the A end product of reaction R2 adsorbed and X_2_ desorbed. Figure 4c shows the DOS for adsorbed MA. Similar to the oxygen vacancy (Figure 4b), there are two occupied states clearly within the bandgap, originating from the surface. Contrary to Figure 4b, Figure 4c shows a third state, with an energy just below the conduction band. This state is also occupied, hence the shift of the Fermi level from Figure 4b to c, indicating that, as a result of reaction R2, an additional electron is donated to the MoO_3_ substrate.

An electron count puts the number of these additional electrons to one (per O vacancy site), as it stems from the oxidation of I^–^, which becomes neutral, as the net charge of an I_2_ molecule is zero. The DOS indicates that the electron transferred to the MoO_3_ is somewhat localized, which would agree with the polaronic localization suggested in ref (58). Moreover, the oxide films used in experiment are likely to show some local inhomogeneity, which further promotes localization of electrons. Such localization of added electrons manifests itself in changed oxidation states, from Mo^6+^ to Mo^5+^, or, depending on the film inhomogeneities to Mo^4+^ or Mo^3+^. This is indeed the case as will be discussed in the next section.

To summarize all the above-described six reactions, oxygen vacancies on the surface of MoO_3_ facilitate the decomposition of the perovskite AX precursors. The decomposition products can in turn create more oxygen vacancies, reducing the involved Mo species, from Mo^6+^ to Mo^5+^. Subsequently, another reaction involving the defective MoO_3_ sites can further reduce to Mo^5+^ or even lower oxidation states. The two redox reactions are accompanied by the oxidation of the involved halide species, with the creation of X_2_. We propose that, upon desorption of the created X_2_ species, the oxygen vacancies become available for further redox reactions, which will in turn further reduce the Mo, making the MoO_3_ surface even more defective and reactive. Overall, the calculated reaction energies (Table 3) indicate that the reactivity of the AX precursors with MoO_3_ decreases in the order of MAI > FAI > MABr > FABr, suggesting that the instability could be mitigated by substituting MA with FA and/or I with Br.

### 2.2 XPS Investigations

To investigate the validity of the DFT calculations, the interaction between MoO_3_ and the AX precursor materials was investigated using XPS to monitor the chemical species at the interfaces and changes in film composition. For this, MoO_3_ films were prepared by thermal evaporation and transferred into a glovebox without air exposure. On top, thin layers (a few nm) of AX were deposited by spin coating a diluted solution (0.05 M), labeled as MoO_3_/AX throughout this article. As reference, thick AX layers (>30 nm) were spin coated on an unreactive surface (PEDOT:PSS) and also measured by XPS.

#### As-Prepared MoO_3_ Substrate

**Figure 5:**
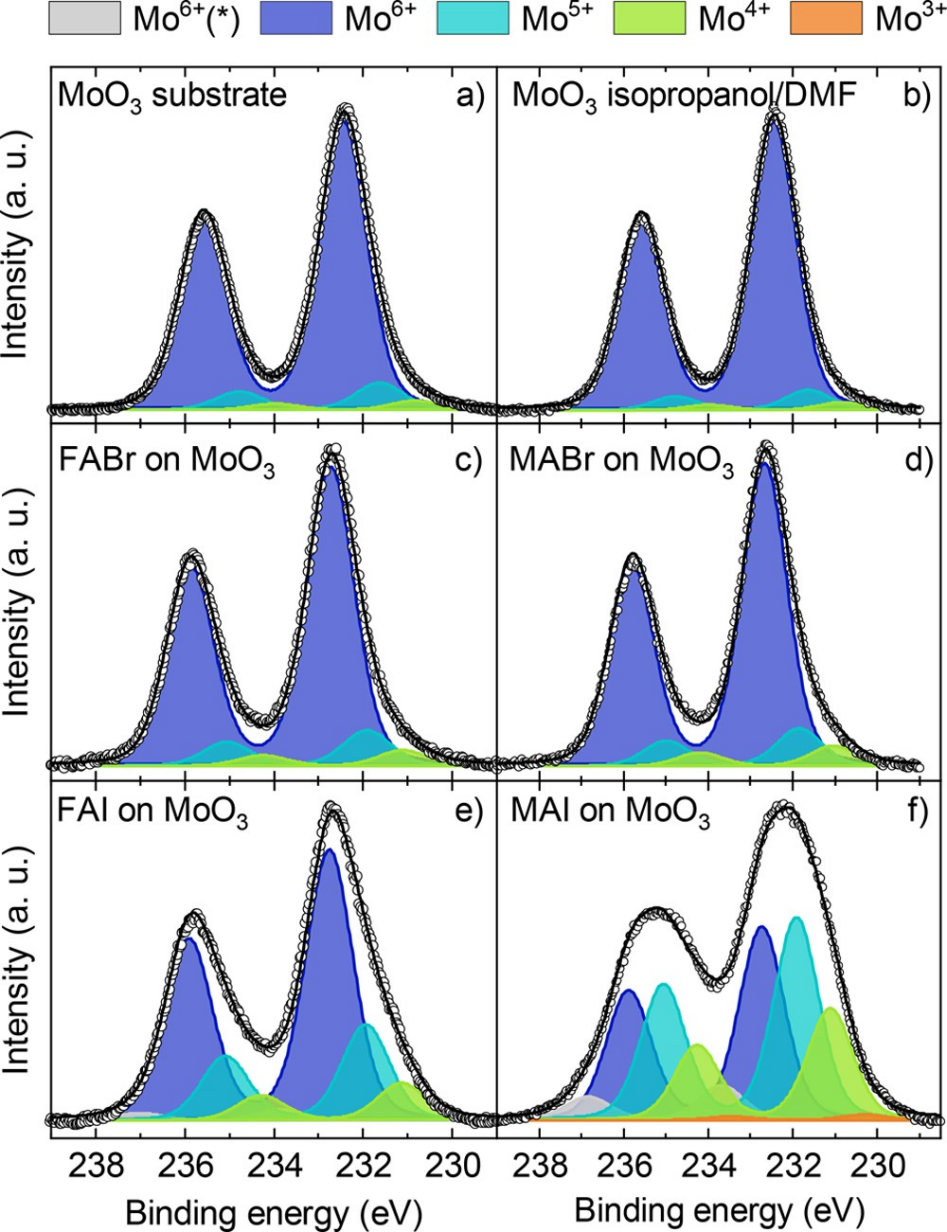
XPS core level measurements of Mo 3d peaks containing fits for the different oxidation states. The samples are (a) the as-prepared MoO_3_ substrate and (b) MoO_3_ substrate after spin coating the pure solvent (isopropanol/DMF) on top, while the remaining graphs show the MoO_3_ substrate covered by an ultrathin layer of (c) FABr, (d) MABr, (e) FAI, and (f) MAI.

**Figure 6:**
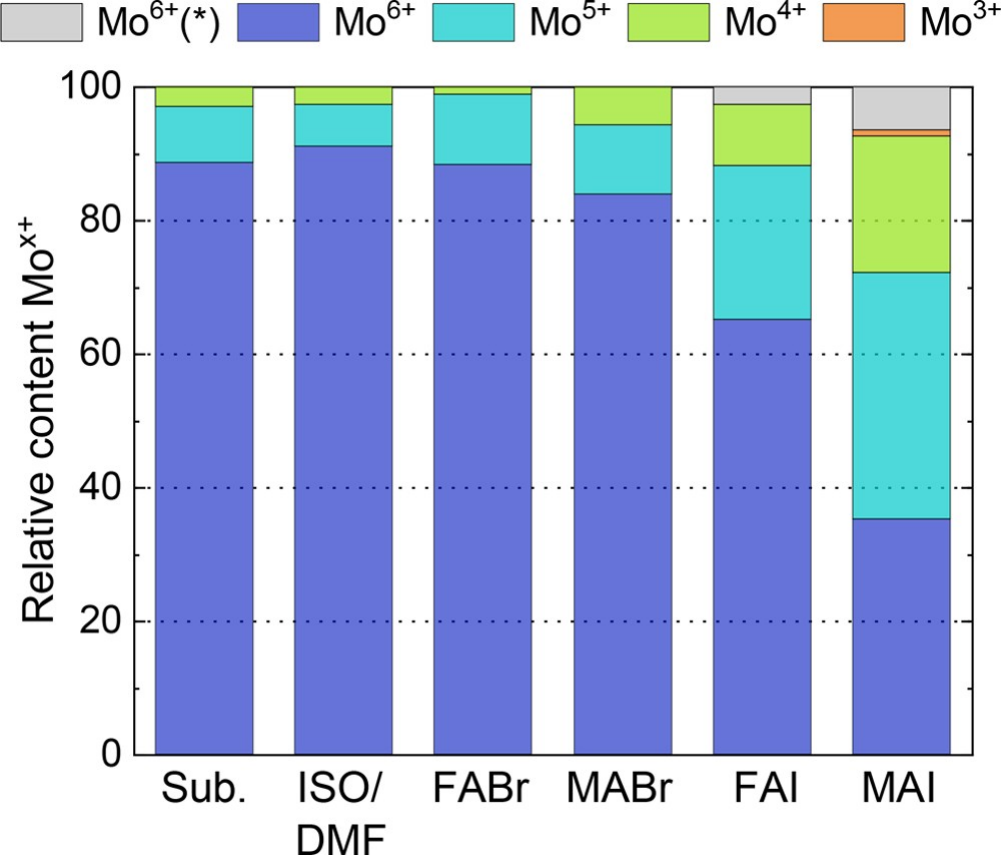
Relative content of Mo oxidation states of the as-prepared MoO_3_ substrate, MoO_3_ after the interaction with the solvent, and after the deposition of an ultrathin precursors layer on top.

First, findings regarding the metal oxide will be discussed, specifically the Mo 3d core level signal. Even though MoO_3_ can be prepared in high purity by evaporation in high vacuum conditions, we find that the core level signal exhibits additional reduced oxidation states besides the expected Mo^6+^ signal, as seen in Figure 5a. Small amounts of Mo^5+^ and Mo^4+^ can be observed in as-prepared MoO_3_, ranging from 2.5–8.5% and 1.5–3.0%, respectively, as summarized Figure 6. The presence of Mo^5+^ and Mo^4+^ indicates the existence of O vacancies in the as-prepared MoO_3_ layer surface that can serve as adsorption sites for the precursor molecules, lowering deprotonation and dissociation reactions of the precursors as suggested by the DFT calculations above. It was further confirmed by the measurement shown in Figure 5b that an interaction with the solvent alone (a mixture of isopropanol and DMF) has no significant effect on the Mo 3d states.

#### Impact of the AX on MoO_3_ Substrate

The calculations shown above suggest that Mo becomes strongly reduced when interacting with the precursor materials. Therefore, the Mo 3d core level XPS spectra for the different MoO_3_/AX interfaces are investigated and are shown in Figure 5. The Mo core level signals, after spin coating the ultrathin layers of the bromide containing precursors, are shown in Figure 5c for MoO_3_/FABr and Figure 5d for MoO_3_/MABr. It can be seen that the XPS spectra look rather similar to the as-prepared MoO_3_ surface, meaning the Mo oxidation states are not significantly altered, as summarized in Figure 6. In contrast, for iodide containing precursors, the reduced Mo oxidation states increase drastically when MoO_3_ gets into contact with either FAI or MAI (Figure 5e,f). Here, the precursor MAI shows a significantly higher ability to reduce Mo^6+^ compared to FAI. As summarized in Figure 6, for FAI more than 60% of the detected Mo atoms remain in the oxidation state 6+, whereas this fraction is less than 40% in the case of MAI. While FAI only increased the amount of 5+ and 4+ states on the surface, MAI also triggered the formation of a small amount of a Mo^3+^ state. It should be noted here, that in order to achieve a good fit to the MoO_3_/MAI and MoO_3_/FAI data, an additional Mo signal had to be added at a binding energy higher than the normal Mo^6+^ signal, labeled as Mo^6+(*)^. This suggests that some Mo atoms are in a chemical environment where more electron density is pulled away compared to the MoO_3_ case. Such a signal has previously been seen and/or used in literature to fit Mo data of thin MoO_3_ films,^45,60^ while in other cases a similar conflict was solved by using a broader full width half max (FWHM) for thinner MoO_3_ films compared to thicker ones or bulk materials.^42,61^ A correlation with results from DFT was unsuccessful; therefore, the feature remains unexplained here.

The stark contrast in interface reactivity between Br and I containing precursors observed in our experiments can be related to the DFT calculations above. As seen in Table 3, the bromide containing precursors are not able to reduce Mo atoms via redox reaction 1 due to a high positive reaction energy of +0.80 eV. The slightly higher reactivity of MABr compared to FABr can be attributed to redox reaction 2, which is, although not favored over simple adsorption of the precursor molecules at the MoO_3_ surface, still more likely to happen for MABr (reaction energy +0.21 eV) compared to FABr (+ 0.39 eV).

In the case of the iodide containing precursors, the negative reaction energy of −0.11 eV in the case of FAI and MAI, enables HI to effectively reduce Mo via redox reaction 1, forming new O vacancies and further increasing the amount of defect sites. Thereby, more surface reactions are triggered that lead to a significant higher amount of reduced Mo oxidation states, compared to the interaction with bromide precursors. The higher reactivity of MAI compared to FAI is likely related to the redox reaction 2: MAI is the only precursor where this reaction is favored over simple adsorption (negative reaction energy of −0.11 eV), leading to I_2_ creation accompanied by additional Mo-reduction. For FAI, on the contrary, redox reaction 2 is not favored, but due to the very small positive reaction energy (+0.10 eV) still likely to occur.

Overall, in good agreement with the DFT calculations above, the XPS Mo core level signals in Figure 5 show vast differences in reactivity of the different precursors with the MoO_3_ surface. The ability to affect the MoO_3_ correlates with differences in reaction energy of the two possible redox reaction routes summarized in Table 3. It can be stated, that the precursor reduction potential toward Mo^6+^ is most pronounced in MAI, followed by FAI, while MABr and FABr do not seem to react with MoO_3_ in a substantial way.

#### Precursor Core Level Signals

**Figure 7:**
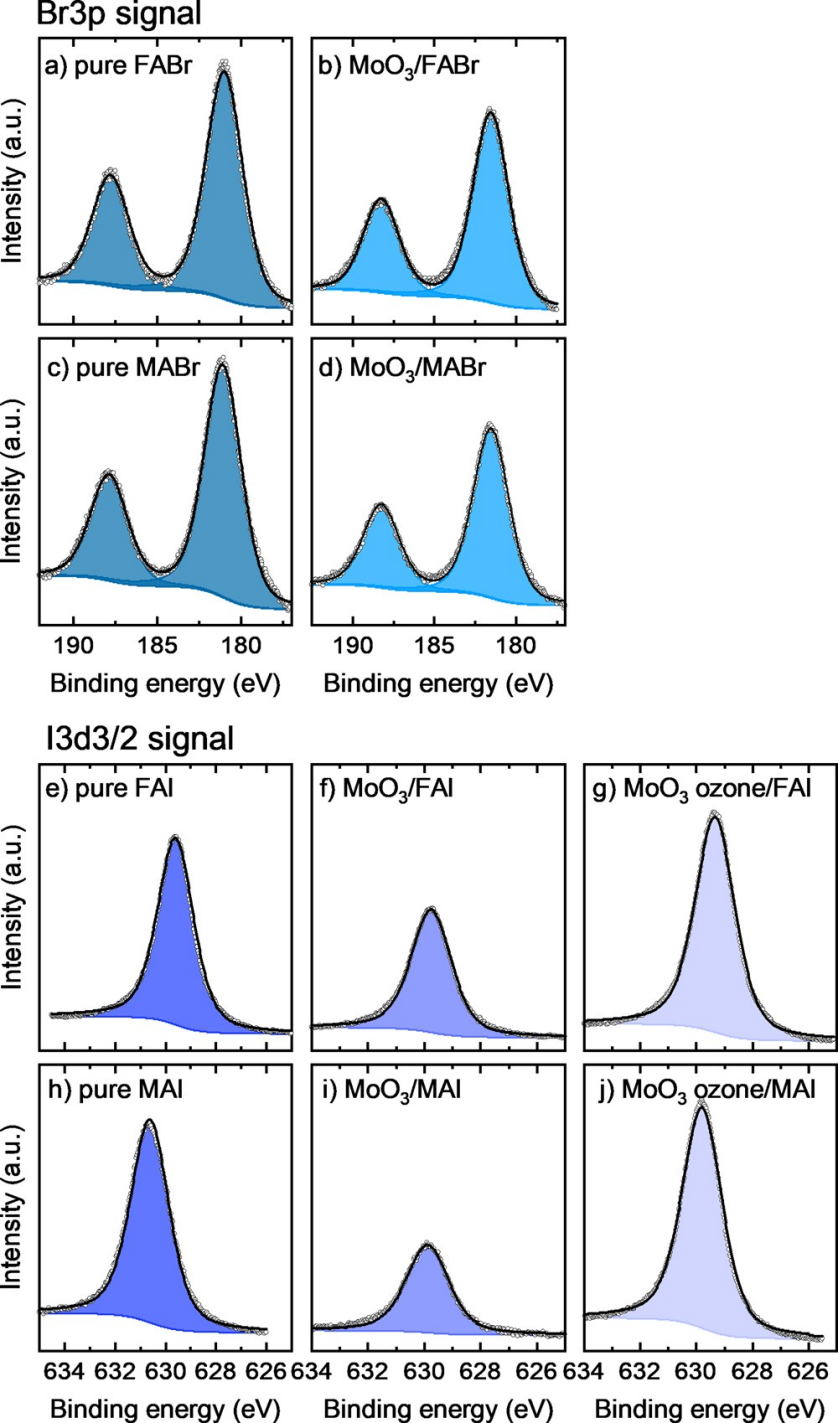
XPS core level measurements. Turquoise shaded fits: Br 3p peaks of (a) an unreacted thick FABr layer, (b) an ultrathin layer of FABr on MoO_3_, (c) an unreacted thick MABr layer, and (d) an ultrathin layer of MABr on MoO_3_. Blue shaded fits: I 3d3/2 peaks of (e) an unreacted thick FAI layer, (f) an ultrathin layer of FAI on MoO_3_, (g) an ultrathin layer of FAI on ozone treated MoO_3_, (h) an unreacted thick MAI layer, (i) an ultrathin layer of MAI on MoO_3_, and (j) an ultrathin layer of MAI on ozone treated MoO_3_.

Next to the effect of the AX perovskite precursors on the metal oxide’s oxidation states, it is also relevant to study the accompanying effect on the AX precursors. To this end, the relative intensities of the precursor specific core level signals of thick (unreacted) AX films are compared to the samples of ultrathin AX films on MoO_3_. Figure 7 shows the halide signals of these pure (thick) AX layers as well as the MoO_3_/AX interfaces. Note that these spectra have been normalized to the intensity of the nitrogen signal to be able to directly compare layers with different thicknesses and therefore different overall signal intensities. This way, a loss of the halide species relative to nitrogen due to redox reactions can be directly seen by changes in signal height. The fits for N 1s are shown in Supporting Information Figure S7. The carbon core levels could not be reliably fitted due to substrate and/or solvent signals superimposing on the signal.

When comparing the bromide signals after contact to MoO_3_ (Figures 7b,d) with the signals from the precursor layers (Figures 7a,c), it is clear that the film stoichiometry (N to Br ratio) is not strongly affected; around 80% of bromide remains present in the samples, as can be seen in Table 4. No additional peaks and no changes in FWHM are observed, which would indicate the presence of additional oxidation states (see the Experimental section for peak fitting procedure). Therefore, no bromide-based decomposition products can be detected on the sample surface. This observation is in excellent agreement with Figure 5, where no significant interaction between Br containing precursors and MoO_3_ was found.

**Table 4:** Halide Content of the Thick Unreacted AX Precursor Layers and the Precursors Spin Coated As Ultathin Layers on MoO_3_ Substrate^a^

	MAI	MoO_3_/MAI	FAI	MoO_3_/FAI	MABr	MoO_3_/MABr	FABr	MoO_3_/FABr
N	1.0	1.0	2.0	2.0	1.0	1.0	2.0	2.0
I	1.1	0.5	1.0	0.6				
Br					0.9	0.7	1.1	0.9

a For MAI and FAI, ozone treated MoO_3_ substrates are included (note, values are normalized to the amount of N on the surface, which is set to be 1.0 for MA and 2.0 for FA).

Since a pronounced reactivity toward Mo was found for iodide containing precursors, more changes can be expected to be observed here. Indeed, a large decrease of the iodide XPS signals is observed for MAI and FAI (compare Figure 7f and i with e and h), suggesting that a volatile iodine species, most likely I_2_ as a product of redox reactions, is formed. This effect is more pronounced in MAI, where only 50% of the iodide species remain on the sample surface, while the loss is slightly less pronounced but still significant in FAI (∼60% of expected intensity found). Since no neutral halide signal is found at higher binding energies, it can be assumed that I_2_ readily leaves the sample surface, as predicted by the DFT calculations.

Overall, XPS shows that the tendency of the materials to form volatile X_2_ follows the order MAI ≫ FAI > MABr > FABr. This trend is in agreement with the previously established ability of the AX precursors to reduce Mo^6+^ to lower oxidation states, as well as the results gained by DFT calculations.

#### Role of Deposition Order

Similar experiments, using a reversed deposition order in an in situ experiment, were also conducted in order to test if MoO_3_ deposited on top of AX leads to comparable results. For this, 2.5 nm of MoO_3_ was evaporated on top of the different 30 nm thick AX layer; the measurements of these AX/MoO_3_ interfaces can be found in the Supporting Information, Figures S5–S7. Intriguingly, the deposition order seems to play only a minor role in the interface reactivity. Compared to the solution processed interfaced presented above, a slightly higher fraction of reduced Mo species was observed in some cases when MoO_3_ was evaporated on top of the precursors (up to 20%, see Figure S5) and the overall decrease in the halide signals is also slightly higher here (up to 10%, Figure S5 and Table S5 in SI). Importantly, the overall trends regarding the reactivity remain the same. The increased reactivity is likely due to the fact, that the evaporated MoO_3_ carries a certain amount of thermal energy when impacting on the precursor surface, since the evaporation temperature of MoO_3_ is above 600 °C.

#### Surface Passivation

**Figure 8:**
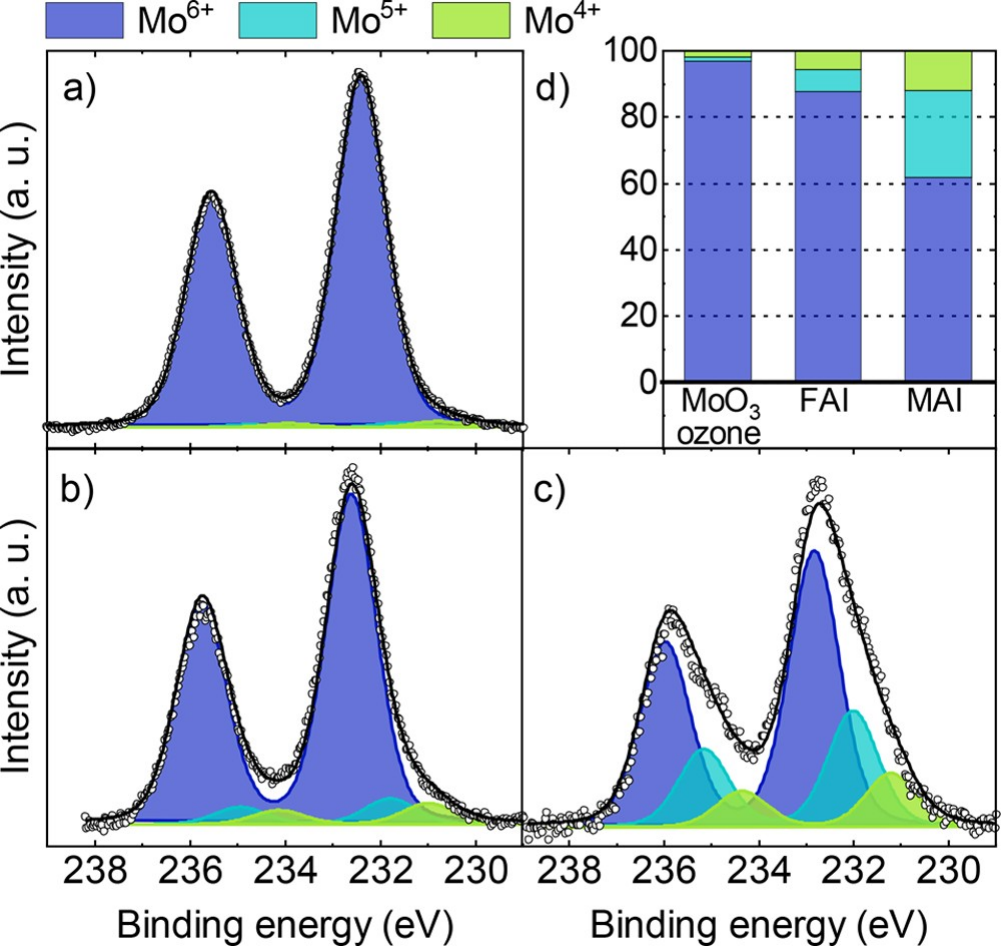
XPS core level measurements of Mo 3d peaks containing fits for the different oxidation states. (a) Mo signals of the ozone treated MoO_3_ substrate. (b,c) Ozone treated MoO_3_ substrate covered with an ultrathin layer of FAI and MAI, respectively. (d) Relative content of Mo oxidation states (extracted from fits in a–c).

In the previous section we found that avoiding iodide containing precursors at the interface to MoO_3_ could be an option to bypass the commonly observed interfacial instability. In addition, the DFT calculations suggest that another possible route could be the elimination of oxygen vacancies. Without these vacancies, the energy barrier of the deprotonation reaction is high and HX should not be generated; hence redox reaction 1 cannot take place. Similarly, for redox reaction 2, O_vac_ are needed as an adsorption site for the halogen to initiate the reaction. Reducing the number of oxygen vacancies on the MoO_3_ surface should therefore decrease the reactivity of the iodide containing precursors with MoO_3_. To verify this assumption, the surface of MoO_3_ layers was UV-ozone treated. Looking at the resulting Mo 3d core level signal in Figure 8a, it is obvious that the amount of lower oxidation states in the ozone treated MoO_3_ substrate is significantly reduced (from 8.4% Mo^5+^ and 2.8% Mo^4+^ in as prepared MoO_3_ to 1.3% Mo^5+^ and 1.8% Mo^4+^ in ozone treated MoO_3_).

On top of these pretreated surfaces, ultrathin layers of either FAI or MAI were spin coated, similar to the experiments presented above in Figure 5. The Mo core level signals in contact with the precursors are shown in Figures 8b,c. It can clearly be seen that upon contact the amount of reduced Mo species is significantly lower compared to the experiments on untreated MoO_3_ layer (see Figure 5e and f). Notably, the amount of reduced Mo oxidation states for MoO_3_(ozone)/FAI is now as low as the bromide containing precursors (FABr and MABr). In case of MAI, now ∼2/3 of the Mo remains in the Mo^6+^ state. Although still a significant reactivity remains, this is an improvement by a factor of 2.

This reduced reactivity is noticeable in the precursor related surface composition as well. The N 1s and I 3d3/2 core levels are presented in Figure 7g,j and in the SI, Figures S5 and S6, while the extracted halide to nitrogen contents are listed in Table 5. Indeed, in case of FAI, no loss in iodide relative to the nitrogen signal is observed, the stoichiometry of the FAI precursor after interacting with the ozone treated MoO_3_ corresponds well with the values obtained for the nondecomposed FAI. In case of MAI on ozone treated MoO_3_, the iodide content remains stable as well, when compared to the unreacted MAI. Although the small amount of reduced oxidation states in Mo suggests that some redox reaction still occurs (most likely redox reaction 1), no loss of iodine was observed anymore. This is, compared to the interaction with untreated MoO_3_, a drastic increase in stability of the MAI precursor.

**Table 5:** Halide Content of the Thick MAI and FAI Precursors Layer and the Precursors Spin Coated As Ultrathin Layers on Ozone Treated MoO_3_ Substrate^a^

	MAI	MoO_3_(ozone)/MAI	FAI	MoO_3_(ozone)/FAI
N	1.0	1.0	2.0	2.0
I	1.1	1.2	1.0	1.1

a Note, values are normalized to the amount of N on the surface, which is set to be 1.0 for MA and 2.0 for FA.

## 3 Conclusions

In this work, we investigate the interaction of MoO_3_ with different perovskite precursors using a combination of DFT calculations and XPS measurements. The calculations indicate that a pristine MoO_3_ surface is unlikely to decompose the perovskite precursors and oxygen vacancies are proposed as reaction centers for such processes. The existence of vacancies in the range of a few percent in as-deposited MoO_3_ is demonstrated via XPS investigations as evidenced by the presence of Mo atoms with reduced oxidation states. We suggest that reaction products of the precursor decomposition may create additional oxygen vacancies, reducing MoO_3_ even further. In addition, we propose that oxygen vacancy sites are also involved in oxidizing the precursor halide molecules directly, adding to the reduction of the oxide. In the proposed scenario, reduction of MoO_3_ is accompanied by oxidation of the halides. Interestingly, both from the calculations and from experiment, I is found to be much more reactive than Br.

XPS measurements show that bromine containing precursors do not significantly affect MoO_3_, while iodide containing organic cations (MAI and FAI) strongly reduce the metal oxide. This is accompanied by a significant loss of iodine, which forms a volatile compound (I_2_). In addition, both DFT calculations and XPS measurements lead to the same order in precursor reactivity, which is MAI ≫ FAI > MABr > FABr. Our results suggest that avoiding I at the interface with MoO_3_ will be an effective way to reduce the interfacial instability known for this metal oxide. In addition, we are able to show that lowering the number of oxygen vacancies by an ozone treatment also leads to an improved stability of the iodide containing precursors on the MoO_3_ surface.

Overall, our investigations on these precursor materials show that the interface reactivity of a metal oxide toward an adjacent perovskite layer is strongly affected by the choice of organic cation and halide, as well as the surface defect density. It is therefore worth not disregarding metal oxide layers, such as MoO_3_, just because of reports of instability toward certain perovskite compositions.

## 4 Computational and Experimental Details

### Computational Details

Density functional theory calculations were performed using the projector augmented wave (PAW) method as implemented in the Vienna Ab-Initio Simulation Package (VASP).^62−65^ The electronic exchange-correlation interaction was described by the functional of Perdew, Burke, and Ernzerhof (PBE) within the generalized gradient approximation (GGA).^66^ Energy and force convergence criteria of 10^–5^ eV and 10^–2^ eV/°A, respectively, were used in all calculations.

MoO_3_ is a layered material that comprises a stack of bilayers, where each bilayer consists of corner-connected and edge-shared MoO_6_ octahedra, and the bonding between the bilayers originates from weak van der Waals forces. Due to the weak interlayer interactions, a single bilayer is sufficient for the study of the MoO_3_ surface. For all the calculations a (3 × 3) bilayer and a vacuum region of 12 Å were used. The calculations were performed with a (2 × 2 × 1) Monkhorst–Pack *k*-point grid and a kinetic energy cutoff of 500 eV. A dipole correction was employed to avoid interaction between periodic images.^67^ For the density of states calculations a (4 × 4 × 1) Monkhorst–Pack *k*-point grid was used along with the DFT+U method as proposed by Dudarev et al.^68,69^ Following the literature, a value of 6.3 eV was used for the *U*–*J* parameter.^70^

The adsorption energies of the perovskite precursors and their dissociation products on the MoO_3_ surface were calculated as
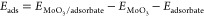
6where *E*_MoO3_*/*adsorbate, *E*_MoO3_, and *E*_adsorbate_ are the DFT total energies of MoO_3_ with adsorbed species, the clean MoO_3_ surface and the adsorbate molecules, respectively. The net atomic charges and bond orders were calculated using the density derived electrostatic and chemical (DDEC6) method.^48,49^ For the charge difference plots, the charge density of the clean MoO_3_ monolayer and that of the free-standing molecules, both in the adsorption geometry, were subtracted from the charge density of the MoO_3_ with adsorbed species.

### Experimental Details

MoO_3_ films were prepared on ITO substrates, onto which 31 nm of MoO_3_ (Alfa Aesar, 99.95% metal basis) layer were evaporated in a vacuum chamber (base pressure: *p* < 7 × 10^–7^ mbar) which is directly attached to the measurement system. A deposition rate of 0.1 Å/s was recorded by a calibrated quartz crystal monitor (QCM) using 4.69 g/cm^3^ as the density for MoO_3_. The MoO_3_ substrates were then transferred under a nitrogen atmosphere into the preparation glovebox, where diluted 0.05 molar precursor solutions were spin coated (spin rate 1500 rpm for 45 s). Afterward, the samples were annealed at 80 °C for 40 min and then transferred under nitrogen atmosphere back into the analysis chamber for XPS measurements

For the thick precursor layers, as well as the reversed deposition order presented in the SI, an aqueous solution of PEDOT:PSS (Clevios P VP Al 4083, Heraeus) was spin coated at 2500 rpm onto ozone-treated inch-sized ITO substrates and annealed at 150 °C for 10 min. The thick films of MAI, FAI, MABr, and FABr (all purchased from Great Cell Solar, purity >99.99%) were prepared via spin coating under nitrogen atmosphere in a glovebox. For that, in case of MAI, FAI, and FABr, 5 × 110 μL of a 0.5 molar precursor solution in 98% isopropanol (Honeywell, CHROMASOLV LC-MS, 99.9%) and 2% DMF (Sigma-Aldrich, HPLC grade, ≥ 99.9%), and in case of MABr a 0.5 molar solution in 75% isopropanol and 25% DMF, were spin coated on top of the PEDOT:PSS using the same conditions and solvent ratios as mentioned above. Afterward, the samples were annealed at 80 °C for 40 min. The inch-sized samples were cut into four pieces and transferred to a vacuum chamber under nitrogen atmosphere. Two of these pieces were investigated directly via XPS. On top of the other two samples, a 2.5 nm MoO_3_ layer was evaporated and measured in situ.

The photoelectron spectroscopy measurements were performed on a custom designed multichamber UHV system at a base pressure of *p* ∼ 10^–9^ mbar, using a Phoibos 100 hemispherical analyzer (Specs). The Fermi edge of gold substrates, cleaned via surface sputtering, was used for calibrating the electron binding energy scale. XPS measurements were done using a Mg Kα X-ray source (*h*ν = 1252.6 eV) at a pass energy of 20 eV for nitrogen, iodine, and bromine peaks. For the investigating the molybdenum signals, a pass energy of 10 eV was used to improve the overall energy resolution of the measurement.

#### Peak Fitting

For investigating the XPS peaks the program XPSPEAK 4.1 was used, using a Shirley background for all element peaks. Parameters for Lorentzian to Gaussian ratio (L:G) and FWHM were kept identical for each specific element (L:G for I 3d_3/2_ = 45, Br 3d = 30, N 1s = 30, and Mo 3d = 23; FWHM (±0.05 eV) for I 3d_3/2_ = 1.75 eV, Br 3d = 2.6 eV, N 1s = 1.65 eV and Mo 3d = 1.26 eV) for all fits. Sample compositions were calculated using the respective peak areas after background subtraction and correction by the respective RSF values (calibrated for the system). Afterward, the received areas of the halide signals were normalized to the areas of the *N* signals, set to be *N* = 1.0 for MA containing precursors and *N* = 2.0 for FA precursor molecules. Relative errors of the stoichiometry values caused by the fitting process and uncertainties in the RSF values are estimated to be <10%.

## Supporting Information Available

Additional DFT results (bond order, net charges, adsorption energies), reaction schemes for the pristine MoO_3_ surface, DOS calculations) as well as additional XPS measurements (reversed deposition order, nitrogen peaks) (PDF)
