# Supplementary material for: Decomposition of Organic Perovskite Precursors on MoO3: Role of Halogen and Surface Defects
Source: ACS Appl Mater Interfaces. 2022 Feb 2;14(30):34208–19. doi: 10.1021/acsami.1c20847 (PMC9353771; doi:10.1021/acsami.1c20847)
Supplement: Supplementary file 1 — am1c20847_si_001.pdf [file am1c20847_si_001.pdf]

# Supporting Information

## Decomposition of organic perovskite precursors on $MoO_3$: role of halogen and surface defects

Sofia Apergi[1,2,§], Christine Koch[3, §], Geert Brocks[1,2,4], Selina Olthof[3*], and Shuxia Tao[1,2*]

[1]Materials Simulation and Modelling, Department of Applied Physics, Eindhoven University of Technology, P.O. Box 513, 5600 MB Eindhoven, The Netherlands

[2]Center for Computational Energy Research, Department of Applied Physics, Eindhoven University of Technology, P.O. Box 513, 5600 MB Eindhoven, The Netherlands

[3]Department of Chemistry, University of Cologne, Greinstraße 4-6, 50939 Cologne, Germany

[4]Computational Materials Science, Faculty of Science and Technology and MESA+, Institute for Nanotechnology, University of Twente, P.O. Box 217, 7500 AE Enschede, The Netherlands

[§] S.A. and C.K. contributed equally to this paper

[§] S.A. and C.K. contributed equally to this paper

* Corresponding Authors Email:

Selina.olthof@uni-koeln.de

S.X.Tao@Tue.nl

### Additional DFT results

***Table S1**. Bond order and net charges of the AX precursors in the gas phase.*

| AX | A-X Bond Order | (X) A Net Charge |
|---|---|---|
| MAI | 0.59 | (-) 0.66 |
| FAI | 0.60 | (-) 0.69 |
| MABr | 0.66 | (-) 0.62 |
| FABr | 0.63 | (-) 0.69 |

***Table S2.*** *Bond lengths (and bond orders inside the parentheses) of the two NH…O bonds between MA/FA and pristine* $MoO_3$.

| MA | FA |
|---|---|
| 2.11 Å (0.08) | 2.15 Å (0.06) |
| 2.22 Å (0.06) | 2.27 Å (0.05) |

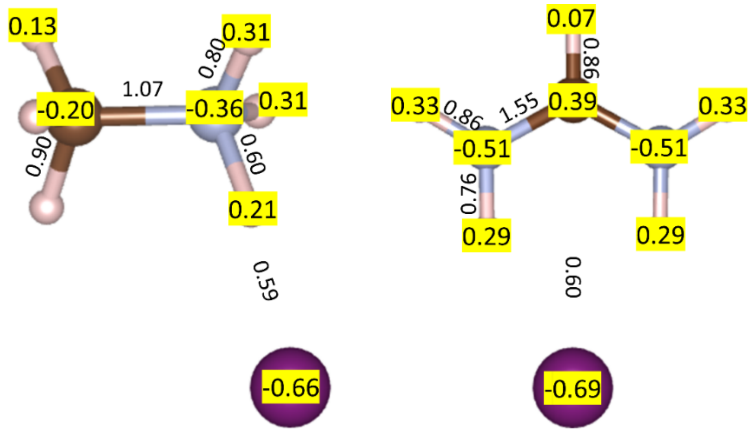


***Figure S1***. *Net atomic charge (yellow) and bond orders for MAI and FAI in the gas phase*.

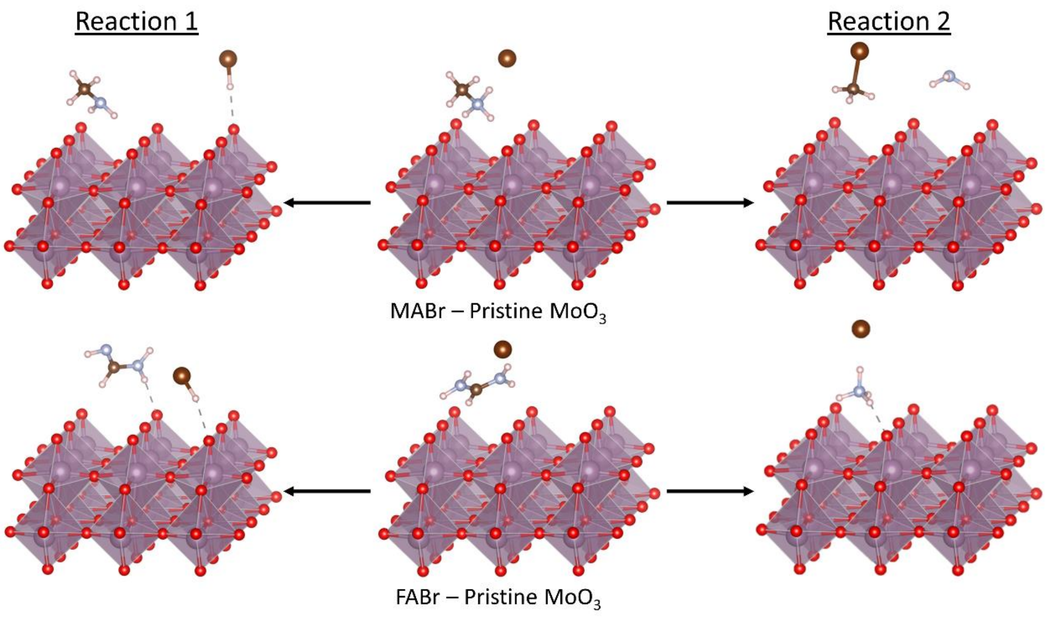


***Figure S2.*** *Atomistic representation of deprotonation (left) and dissociation (right) reactions of MABr (top) and FABr (bottom) on the pristine* $MoO_3$. *HCN (product of reaction 2) is not presented as it leaves the surface of* $MoO_3$.

***Table S3.*** *Adsorption energies of the precursor molecules and of the products of the dissociation reactions on the pristine and defective $MoO_3$ surface.*

| Adsorbent | Adsorption Energy (eV) | |
|---|---|---|
| | Pristine $MoO_3$ | Defective $MoO_3$ |
| MAI | -0.87 | -1.63 |
| FAI | -0.79 | -1.30 |
| MABr | -0.62 | -1.65 |
| FABr | -0.58 | -1.34 |
| Methylamine | -0.25 | -1.05 |
| Formamidine | -0.22 | -1.47 |
| HI/HBr | -0.04/-0.04 | -0.48/-0.43 |
| $NH_3$ | -0.11 | -0.92 |
| $CH_3I$/$CH_3Br$ | -0.09/-0.04 | -0.10/-0.09 |
| $NH_4I$/$NH_4Br$ | -0.89/-0.58 | -1.61/-1.57 |
| HCN | -0.04 | -0.46 |

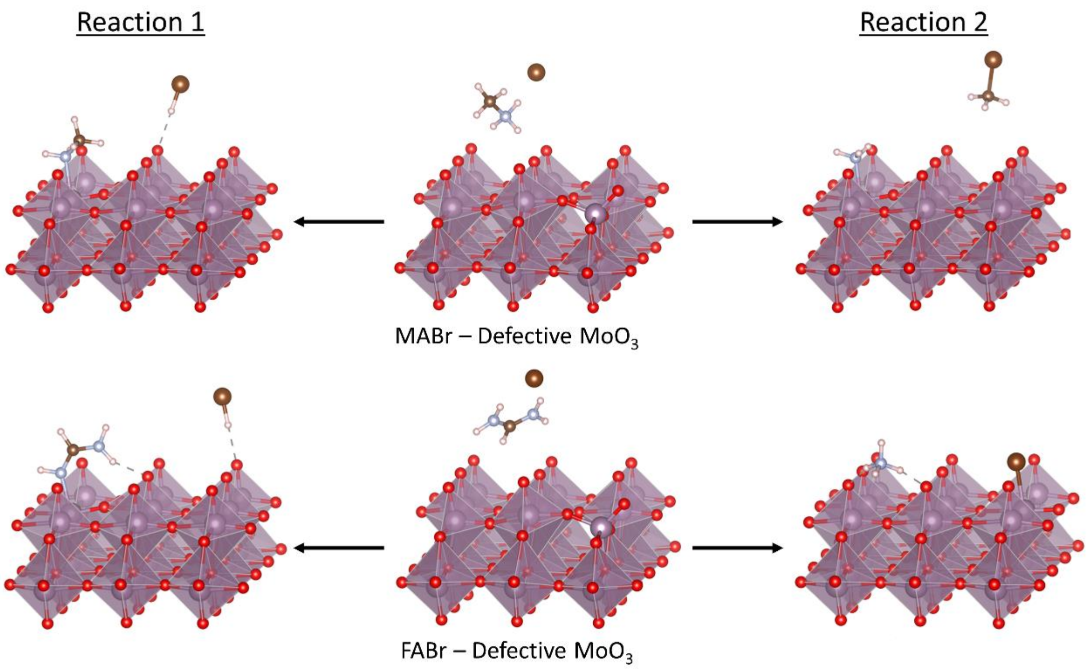


***Figure S3***. *Atomistic representation of deprotonation (left) and dissociation (right) reactions of MABr (top) and FABr (bottom) on the defective $MoO_3$. HCN (product of reaction 2) is not presented as it leaves the surface of $MoO_3$.*

***Table S4***. *Calculated net atomic charge of HX adsorbed on the pristine $MoO_3$ surface.*

| Adsorbent | H | X |
|---|---|---|
| HI | 0.13 | -0.07 |
| HBr | 0.20 | -0.18 |

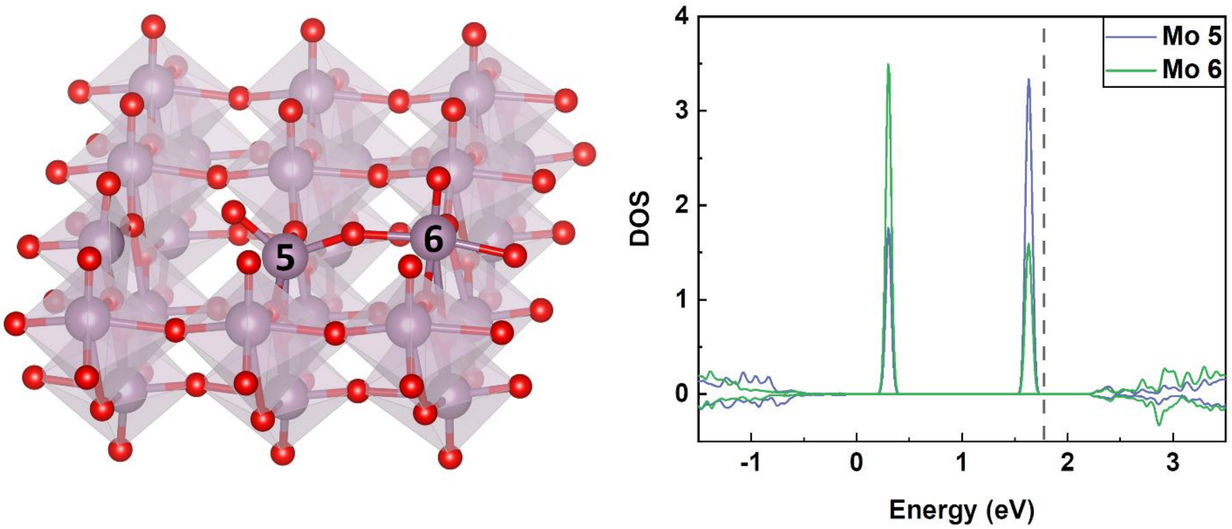


***Figure S4***. *Spin polarized density of states for the Mo atoms 5 and 6 as shown in the corresponding atomistic structure. The zero of energy is set at the valence band maximum. The dashed line denotes the highest occupied state.*

## Additional experimental data

### Role of deposition order on XPS experiments

In addition to the experiments shown in the main article, also the reversed deposition order was probed in an in-situ experiment to test if $MoO_3$ deposited on top of AX leads to comparable results. For this, 2.5 nm of $MoO_3$ was evaporated on top of the different 30 nm thick AX layer; the measurements of these AX/$MoO_3$ interfaces can be seen in Figures S4 – S6. Overall, the same trend in reactivity is found, though the degradation seems to be slightly enhanced as discussed in the main article. However, in contrast to the interface presented in the main article, no additional $Mo^{3+}$ signal was observed for MAI interaction with $MoO_3$.

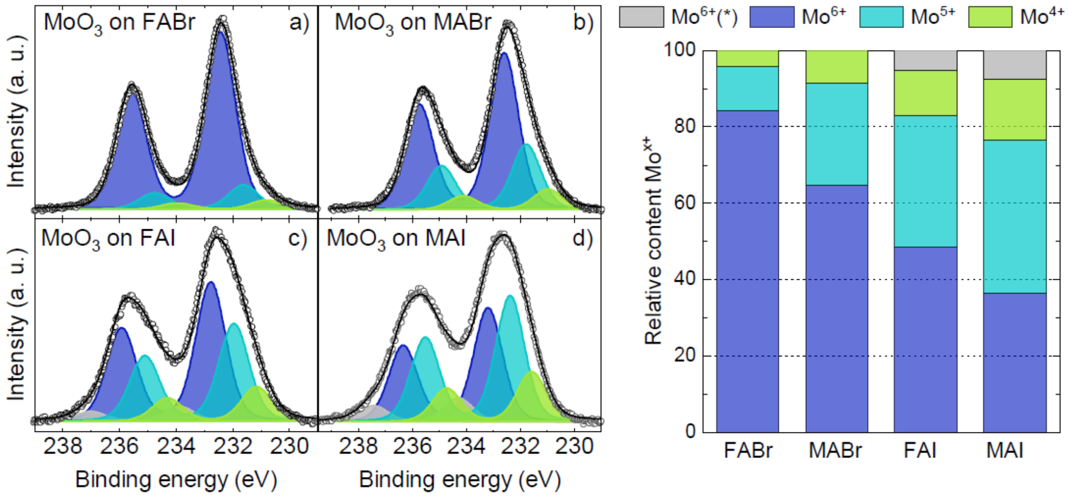


***Figure S5***. *XPS core level measurements of Mo3d peaks containing fits for the different oxidation states for the samples with reversed deposition order AX/$MoO_3$. Here 2.5 nm of $MoO_3$ were deposited on thick precursor films: a) on FABr, b) on MABr, c) on FAI, and d) on MAI. The right hand image shows the relative content of Mo oxidation states extracted from the fits in a) - d).*

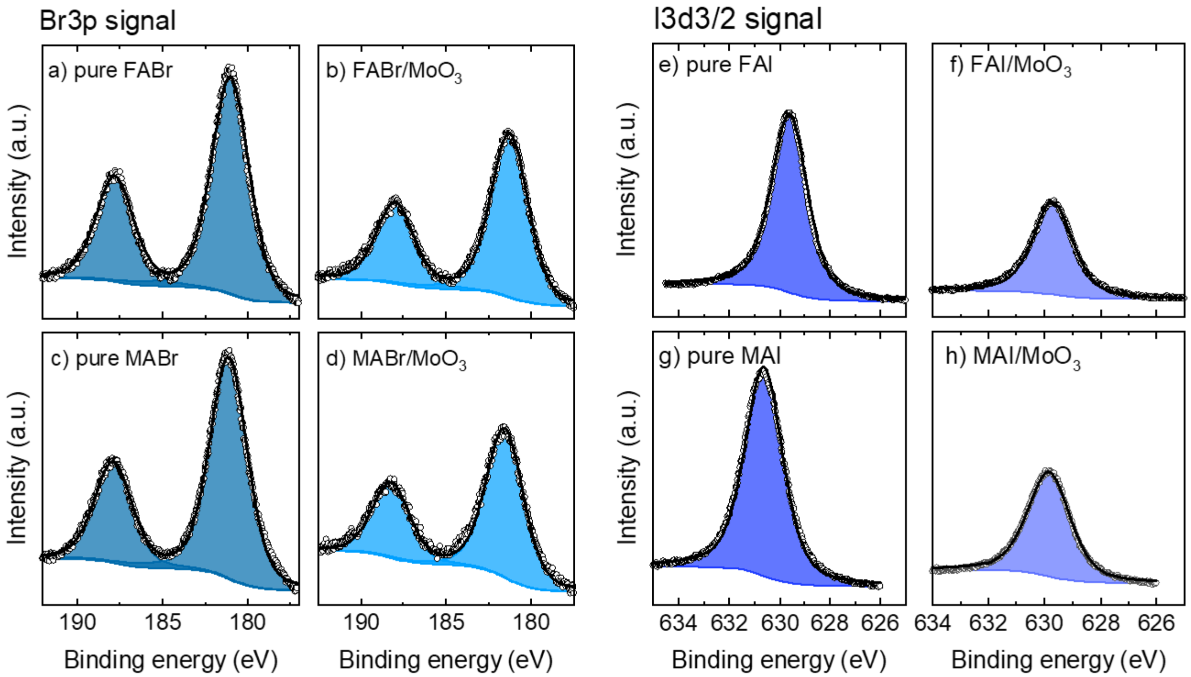


***Figure S6.*** *XPS core level measurements of the halide signals for the samples with reversed deposition order AX/$MoO_3$. Turquoise shaded fits: a) and b) show the Br3d peaks of an unreacted thick FABr layer before and after deposition of a 2.5nm thick $MoO_3$ layer on top. c) and d) show a similar experiment for MABr. Blue shaded fits: e) and f) show the I3d3/2 peak of an unreacted thick FAI layer before and after deposition of a 2.5nm thick $MoO_3$ layer on top. g) and h) show a similar experiment for MAI.*

**Fit of Nitrogen peaks**

To determine the precursor stoichiometry, the peak areas of the halides as well as the nitrogen signals are needed. As seen in Figure S7, the N1s signal is partially overlapping with the Mo 3p signal that makes the peakfitting more challenging when the precursor is in contact to $MoO_3$. Therefore, it cannot be ruled out that during the precursor/$MoO_3$ interaction a small amount of reduced / neutral N species (that would cause an additional N1s signal at lower binding energies) occur. No change in FWHM of the fitted N1s peaks is observed through, so we are nevertheless quite confident that no significant reduced precursor species were present in the samples.

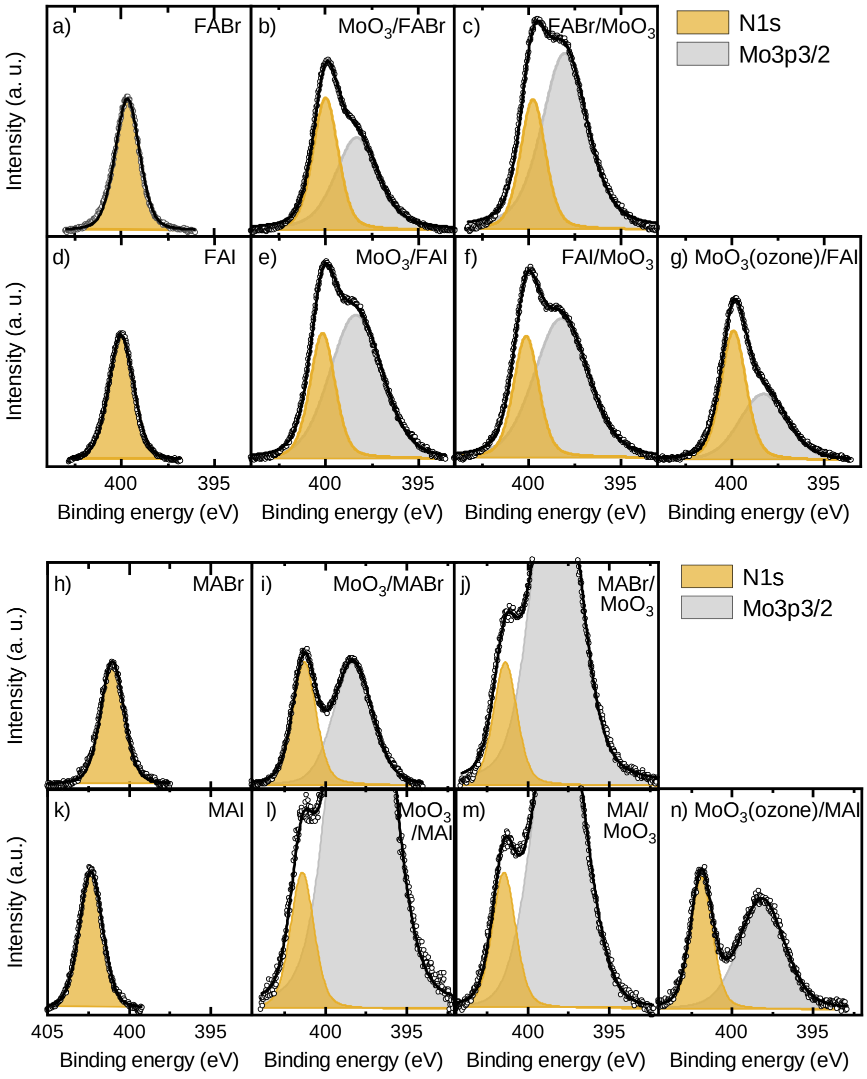


***Figure S7***. *XPS core level measurements of nitrogen 1 s signals of all samples presented in the main article as well as the SI. Top: a) thick layer FABr, b) ultra-thin layer of FABr on top of $MoO_3$, and c) FABr with a 2.5 nm $MoO_3$ layer on top. d) to f) show similar results for FAI, while g) presents the additional measurement of FAI on top of ozone tread $MoO_3$. Bottom: h) thick layer MABr, i) ultra-thin layer of MABr on top of $MoO_3$, and c) MABr with a 2.5 nm $MoO_3$ layer on top. k) to m) show similar results for MAI while n) presents the additional measurement of MAI on top of ozone tread $MoO_3$. Orange shaded peaks represent the N signal while the grey one indicates the overlapping $Mo3p_{3/2}$ signal. The respective nitrogen signals of MA or FA compounds all shown here as the same height, since the signals have been normalized to the same intensity in order to remove the influences of effective layer thickness / signal intensity.*

***Table S5.*** *Halide content of the thick AX precursors after reacting with an 2.5 nm thick $MoO_3$ layer evaporated on top (values are normalized to the N amount, which is set to be 1.0 for MA and 2.0 for FA).*

| | | MAI /$MoO_3$ | FAI /$MoO_3$ | MABr /$MoO_3$ | FABr /$MoO_3$ |
|---|---|---|---|---|---|
| N | | 1.0 | 2.0 | 1.0 | 2.0 |
| I | | 0.6 | 0.5 | - | - |
| Br | | - | - | 0.6 | 0.8 |
